# Changes on the conformational and functional properties of soybean protein isolate induced by quercetin

**DOI:** 10.3389/fnut.2022.966750

**Published:** 2022-07-22

**Authors:** Yating Zhang, Ruiyang Hou, Beibei Zhu, Guangwei Yin, Jian Zhang, Wenqi Zhao, Junxi Zhang, Taoran Li, Zifan Zhang, Hongwu Wang, Zheng Li

**Affiliations:** ^1^School of Public Health and Health Sciences, Tianjin University of Traditional Chinese Medicine, Tianjin, China; ^2^College of Chinese Medicine Pharmaceutical Engineering, Tianjin University of Traditional Chinese Medicine, Tianjin, China; ^3^Key Laboratory of Industrial Fermentation Microbiology, Ministry of Education, Tianjin University of Science and Technology, Tianjin, China

**Keywords:** soybean protein isolate, quercetin, multi-spectroscopic techniques, molecular docking, interaction

## Abstract

The conformational changes and functional properties of SPI induced by quercetin was investigated via fourier transform infrared (FTIR) spectroscopy, fluorescence spectroscopy, circular dichroism (CD) spectroscopy and molecular docking. A decrease in the fluorescence intensity and a blue shift in the maximum wavelength were observed due to the binding process with fluorescent residues. The analysis of Stern-Volmer equation showed that the fluorescence quenching induced by quercetin took the form of static quenching, and the binding stoichiometry between SPI and quercetin was 1:1. The values of ΔH and ΔS were both positive illustrating that hydrophobic interaction was the primary binding force between quercetin and SPI. Results of FTIR and CD indicated that the binding with quercetin changed the secondary structure of SPI, resulting in a partially unfolded and more flexible structure. SDS-PAGE confirmed there was no covalent interaction between the two constituents. Molecular docking demonstrated that there were stable configurations and high matching degrees in both 11S and 7S proteins with quercetin via hydrogen bonds and hydrophobic interactions. Meanwhile, modification by quercetin enhanced the foaming and emulsifying capacities of SPI. These findings might provide theory reference for elucidation the mechanism of polyphenols-proteins interaction and development of related food additive products in future.

## Highlights

1. A systematic study is pressly needed on changes in structure and function of SPI with quercetin.
2. Fluorescence quenching induced by quercetin took the form of static quenching.
3. The binding stoichiometry between SPI and quercetin was 1:1.
4. SPI-quercetin complex binded stably by hydrogen bonds and hydrophobic interactions.
5. Binding with quercetin led a more flexible structure and better functional properties of SPI.

## Introduction

Soy protein isolated is known as high-quality protein and widely used in food industry due to its excellent functional properties, high nutritional value, and low cost (1). However, the compact globular structure of SPI leads to the poor solubility and emulsifying properties. And SPI is characterized by weak electrostatic and spatial attraction, and it usually forms unstable emulsions (1, 2). It is easily affected by the processing conditions, such as low pH, high temperature, and ionic environment, which greatly restricted its application in actual production. Many strategies have been made to improve the functional properties of soy protein, including physical methods (3, 4) and chemical methods (4, 5) as well as (6). Recently, It was found that the non-covalent interaction of polyphenols with whey protein could cause the exposure of tryptophan residues, the unfolding of proteins and the decrease of the surface hydrophobicity (7). Consequently, considerable improvement of the functional properties was observed including digestibility, solubility, and foaming capacity. According to Yaohui et al., hydrophobic force and hydrogen bonds were the main interaction forces between SPI and EGCG. Due to the high affinity of EGCG, the structure of SPI became looser and exposed more active groups, thus leading to an improvement of the foaming, emulsifying, and antioxidant properties (8).

Quercetin belongs to polyphenols, which is abundant in numerous fruits and vegetables such as celery, parsley, onions, and blueberry (9). A number of health benefits have been reported for quercetin, including antioxidant (10), anti-inflammatory (11), and anti-aging properties (12). The antioxidant function of quercetin is mainly reflected to enter cells, occupy oxidative damage, and save dying cells. In addition, it is believed that it works mainly by destroying cell structures (13, 14). Mechanistic studies on anti-aging properties revealed that quercetin alleviated senescence by promoting the proliferation of cells and restoring the heterochromatin architecture in WS hMSCs (12). Moreover, quercetin has shown promising antiviral properties, cardioprotective effects (15) as well as potential prophylactic and therapeutic effects against cancers due to its non-mutagenic properties and relatively low toxicity (16). The chemotherapeutic results of quercetin have been confirmed for many human cancers cells, for instance, colorectal cancer (17). Although quercetin have so many biological activities, the solubility is extremely low due to its high hydrophobicity, which greatly limits the application in food and biomedical industries In aqueous solution, the solubility of quercetin decreased with increasing temperature and pH, especially at alkaline pH (18). Additionally, its chemical instability, permeability and oral bioavailability are also poor, and it swiftly exits the circulation before reaching the target organs (19, 20). Recently, researchers developed some stabilization strategies to improve the solubility and biological activity of polyphenols, via the formation of hydrogels, microencapsulation (16), self-assembling polymeric micelles (21), and structural modification such as glycosylation, acylation (22). Although there are several options to address the low applicability of polyphenols, the disadvantages still exist, such as the process of microencapsulation is complicated and during emulsifying, high temperatures in thermal gelation might destroy their bioactivities (23). Current studies have shown that the formation of complexes with other substances such as proteins and polysaccharides is a good choice to make full use of quercetin (24–26). The construction of the SPI-EGCG complex could also improve the gelation rates of protein significantly (27). As compared with SPI alone, the gel formed by the composite had a better network structure, mechanical strength, water holding capacity, and rheological properties (27). Studies revealed that the interaction force of EGCG with 11S and 7S proteins was hydrogen bonding, electrostatic interaction and hydrophobic interaction (10). It has been found that the biological effects of polyphenols could be affected to varying degrees by the presence of protein, in turn, the structural and functional properties of protein has also been influenced by polyphenols. Yang et al. (28) prepared binary complexes with EGCG and 11S/7S proteins that could withstand a certain ionic strength, meanwhile, the physicochemical and functional properties of SPI was also improved. Wang et al. found the stability and radical scavenging activity of quercetin could be improved when used soy protein isolated (SPI) particles after cold-gelation as a protective carrier (29). Ao et al. compared the strength of the interaction between SPI and food polyphenols and found the SPI-polyphenols binding patterns were closely related to the variety of polyphenols (30).

To our knowledge, the interaction of EGCG and various proteins has attracted considerable attention of various researchers and been well-understood. Nevertheless, more systematic study about the structural changes and functional properties of SPI leading by quercetin is necessary. Based on these scientific findings, current research was conducted to characteristic the conformational changes and interaction mechanism of SPI induced by quercetin via a series of spectroscopy methods and molecular docking and the improvement on the functional properties of SPI-quercetin complex. The study aims to get further insights into the effect of polyphenols on the structural and functional properties of SPI, thus, offering as a reference for the development of novel protein additives with good functional properties and improved health benefits in future.

## Materials and methods

### Materials

Quercetin hydrate (HEOWNS, purity ≥99%) was purchased from (Tianjin Heowns Biochem LLC, Tianjin, China), and sodium hydroxide (purity ≥96%) and sodium chloride (purity ≥99.5%) were purchased from Tianjin Jiangtian Chemical Technology Co., LTD (Tianjin, China). Phosphoric acid (purity ≥85%), methanol (purity ≥99.5%) and ethanol (purity ≥99.7%) were purchased from Tianjin Fengchuan Chemical Reagent Technology Co., LTD (Tianjin, China). SurePAGE, Bis-Tris, 10 ×8 (C35222108, 4–12%, 12 wells, 10/pk), Tris-MOPS-SDS Running Buffer Powder (C31382105) and PAGE-MASTER Protein Standard Plus (C2106004) were purchased from GenScript (860 Centennial Ave., Piscataway, NJ 08854, USA). Dimercaptoethanol (purity 99%), Coomassie Brilliant blue R250 (CAS:6104-59-2, Assay ≥90%) and Bromophenol blue (CAS:115-39-9) were purchased from biosharp (Labgic Technology Co., Ltd., Hefei, Anhui Province, China).

### Preparation of soybean protein isolate—quercetin conjugates

Soybean protein isolate was extracted from low-denature defatted meal using alkali solution and acid precipitation method (31) and freeze-dried to powders. The freeze-dried soybean protein isolate powders were fully dissolved in deionized water at a concentration of 1.0 × 10^−4^ mol/L. Quercetin solution was prepared in ethanol at the concentration of 1.0 × 10^−3^ mol/L. Afterwards, The quercetin solution was slowly added to 30 ml SPI solutions, and vortexed for 1 min. The molar ratios of SPI and quercetin were set as 1:0, 1:0.2, 1:0.4, 1:0.6, 1:0.8, 1:1.

### Binding rate analysis

To provide binding information between SPI and Que, binding rate of SPI-quercetin (%) were determined according to the method of Liu et al. (32) with slight modification. All samples were adjusted to pH 4.60 to precipitate the protein-quercetin complexes. The samples were then centrifuged for 5 min at 13,000 rpm, and the absorbance of the supernatant was measured at 373 nm using a UV-Vis spectrophotometer. A standard curve was used to calculate the concentration of quercetin in the solution. Binding rate of SPI-quercetin was calculated using equation:


(1) $$\begin{array}{l} Binding\;rate\;\left(\%\right)=\left(1-\frac{C_{free}}{C_{all}}\right)\times 100\% \end{array}$$

### Intrinsic fluorescence spectroscopy

The fluorescence determination at different temperatures (298K, 308K, and 318K) was carried out using the fluorescence spectroscopy (33). Set the excitation wavelength at 280 nm, the slits of the excitation and emission wavelengths at 5 nm, and the scanning speed at 1,500 nm/min. The emission spectra were adjusted between 300 and 500 nm. Blanks were subtracted from SPI aqueous solutions and deionized water. The following has been determined based on Stern-Volmer analysis:


(2) $$\begin{array}{l} \frac{F_{0}}{F}=1+K_{q}\tau_{0}\left[Q\right]=1+K_{SV}\left[Q\right] \end{array}$$

Here, F_0_ represents fluorescence intensities of the protein solution without quercetin, and F represents fluorescence intensities of the protein with different ratios of quercetin; kq is the bimolecular quenching rate constant; τ_0_ is the average lifetime of the complexes without quercetin (the fluorescence lifetime of tryptophan is 3 × 10^−9^; [Q] is the concentration of the quercetin in system; K_SV_ is the Stern-Volmer quenching constant.

The binding parameters between quercetin and protein can be analyzed by the logarithmic equation:


(3) $$\begin{array}{l} \log\left(\frac{F_{0}-F}{F}\right)=\log K_{a}+n\log\left[QT\right] \end{array}$$

Here, K_a_ represents to the binding constant between quercetin and SPI; n is the binding stoichiometry.

Determined the non-covalent interaction of SPI-quercetin binding from thermodynamic parameters, which were calculated by the Van't Hoff equation:


(4) $$\begin{array}{lll} \ln\;K_{a} & = & -\frac{\Delta H}{RT}+\frac{\Delta S}{R} \end{array}$$

(5) $$\begin{array}{lll} \Delta G & = & \Delta H-T\Delta S \end{array}$$

Here, *R* is the gas constant [8.314 J•(mol•K)^−1^]; *T* is the absolute temperature.

### Fourier-transform infrared (FTIR) spectroscopy

Fourier transform infrared spectroscopy (FTIR) was used to monitor the conformational changes of SPI before and after binding with Que. All samples were prepared according to Section Preparation of Soybean Protein Isolate—Quercetin Conjugates and freeze-dried to powders. A prefabricated sample powder (1 mg) was mixed with KBr (200 mg) and compressed into a transparent flake. The spectrometer (Bruker, Germany) was applied to get FTIR spectra at 4 cm^−1^ resolution, and scanned 64 times for each group at 25 °C, against a background spectrum recorded from the KBr pellet (34).

### Circular dichroism (CD) spectroscopy

The CD spectra of SPI samples with the absence and presence of quercetin at different ratios were measured using a circular dichromatic spectrometer (J-810, Japan). The CD spectra scanning was in the far-UV region (190–250 nm) at room temperature in a 1 cm length quartz cuvette. The scanning speed and bandwidth were set to 50 nm/min and 1.0 nm, respectively. Three parallel groups of samples are required for all samples (35). The content of secondary structures of SPI was calculated with CDSSTR software supported by CDPro software package.

### Sodium dodecyl sulfate-polyacrylamide gel electrophoresis (SDS-PAGE)

Effect of different ratios of quercetin on the molecular properties of soybean protein was evaluated using SDS-PAGE electrophoresis according to method of Wu et al. (36) with minor modifications. Briefly, the samples were diluted 1:1 with the (Laemmli) sample buffer (0.0625 mM Tris-HCl, 2% SDS, 10% glycerol, 5% 2-mercaptoethanol, 0.0025% bromophenol blue), and then heated the systems at 95 °C for 3 min. The aliquots were loaded on 4% stacking gel and 12% separating gel, adjusted the voltage, and sustained 150 volts for 1 h. The gels were stained with Coomassie Brilliant Blue R-250 dye liquor overnight after electrophoresis (5% methanol, 7.5% acetic acid) for 30 min. Subsequently, the gels were distained with decolorizing solution (acetic acid: deionized water = 3:37) and observed by gel imaging analysis system.

### Molecular docking

A docking compound, quercetin, was obtained from the PubChem database (https://pubchem.ncbi.nlm.nih.gov/). After hydrogenation, optimization, and energy minimization, the structural information was imported into the Schrodinger software and used as a ligand. Crystal structure of SPI target protein downloaded from protein database (http://www.rcsb.org/). Since SPI is primarily composed of 7S and 11S globulin, two target proteins were selected. The protein structures were processed using the Maestro 12.7 platform. The protein preparation wizard was used to process the proteins, to remove crystallization waters, to add hydrogen atoms that were missing, and to repair the bonds and peptides. Lastly, optimize structures and minimize energy consumption. Glide of Schrodinger Maestro was used for molecular docking. Using LigPre's default settings, quercetin was prepared, and then imported into Glide to generate active sites in the SPI grid. The SP method was used for molecular docking and screening (37, 38).

The interactions between the compound and the target protein were analyzed, and the interaction modes between the compound and the protein residues were obtained, including hydrogen bonds, π-π interactions, and hydrophobic interactions, etc. The docking score of compounds was used to predict whether the compound to be screened had similar activity to the positive compound.

### Emulsifying activity index (EAI) and emulsifying stability index (ESI)

The Emulsifying activity index (EAI) and emulsifying stability index (ESI) were measured according to the method of Yating Zhang et al. with slight modification (1). The complexes of SPI and quercetin with molar ratio of 1:0.6 were prepared according to Section Preparation of Soybean Protein Isolate—Quercetin Conjugates and mixed with soybean oil (3:1, v/v). The mixture was homogenized at 15,000 rpm for 1 min and 50 μL of the coarse emulsions were mixed in 5 mL of 0.1% (w/v) SDS solution immediately. The absorbance was measured at 500 nm. The EAI and ESI were obtained using the following formulas:


(6) $$\begin{array}{l} EAI\left(m^{2}/g\right)=\frac{2\times 2.303\times A_{0}\times 100}{C\times L\times \theta \times 10000} \end{array}$$

(7) $$\begin{array}{l} ESI\left(\%\right)=\frac{A_{t}}{A_{0}}\times 100 \end{array}$$

Here, A_0_ is the absorbance of the coarse emulsion at 0 min after homogenization. C is the concentration of protein (mg/ml), L is the optical path length of the colorimetric dish, and θ is the oil proportion of the emulsions. A_t_ is the absorbance of the emulsions measured at 5, 10, 20, and 30 min, respectively.

### Foaming capacity (FC) and foaming stability (FS) index

Measurement of Foaming capacity (FC) and Foaming stability (FS) was following the method described by Yaohui You et al with slight modification (8). SPI and SPI-quercetin complexes (100 ml) were whipped by a high speed dissolvers (IKA T25, Instrument Co., LTD, German) at 10,000 r/min for 1 min. The volume of foam was recorded at 0, 10, 20, 30, 40, 50 min, respectively. The FC and FS were calculated as follows:


(8) $$\begin{array}{lll} FC\left(\%\right) & = & \frac{V_{1}-V_{0}}{V_{0}}\times 100 \end{array}$$

(9) $$\begin{array}{lll} FS\left(\%\right) & = & \frac{V_{t}}{V_{1}}\times 100 \end{array}$$

Here, V_0_ is the beginning volume of the mixture, V_1_ is the volume after high speed dispersion (0 min), and V_t_ is the volume after different quiescence time.

### Statistical analysis

All the experiments were conducted in triplicate and the results were expressed as mean ± standard deviation. All data were subjected to analysis of variance (ANOVA) and Duncan's multiple range test using SPSS at the significance level of *P* < 0.05. The figures in this article were created using Origin 2019b.

## Results

### Binding rate of SPI-quercetin

**Figure 1:**
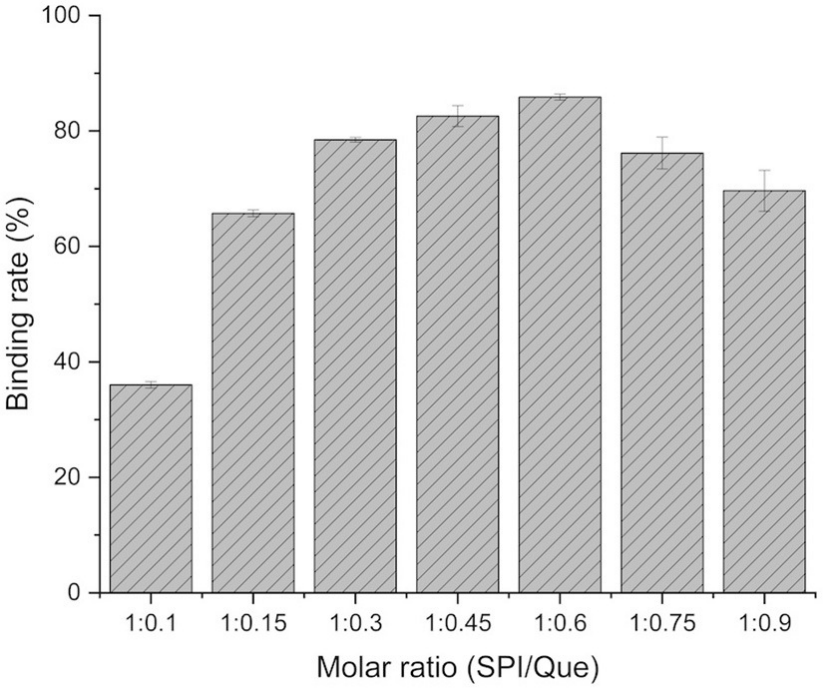
The binding rate (%) of SPI-quercetin (Que) at different ratio.

Result of SPI-quercetin binding rate is shown in Figure 1. The binding rate ranged from 36.03 to 85.86% and mainly concentrated in 70–82%. When the mole ratio of SPI to quercetin was 1:0.6, the binding rate reached the highest level (85.86%). This result indicated that there was an effective combination between SPI and Que. Furthermore, the research of Antonio et al. showed similar results to our findings (39).

### Fluorescence spectrum analysis

**Figure 2:**
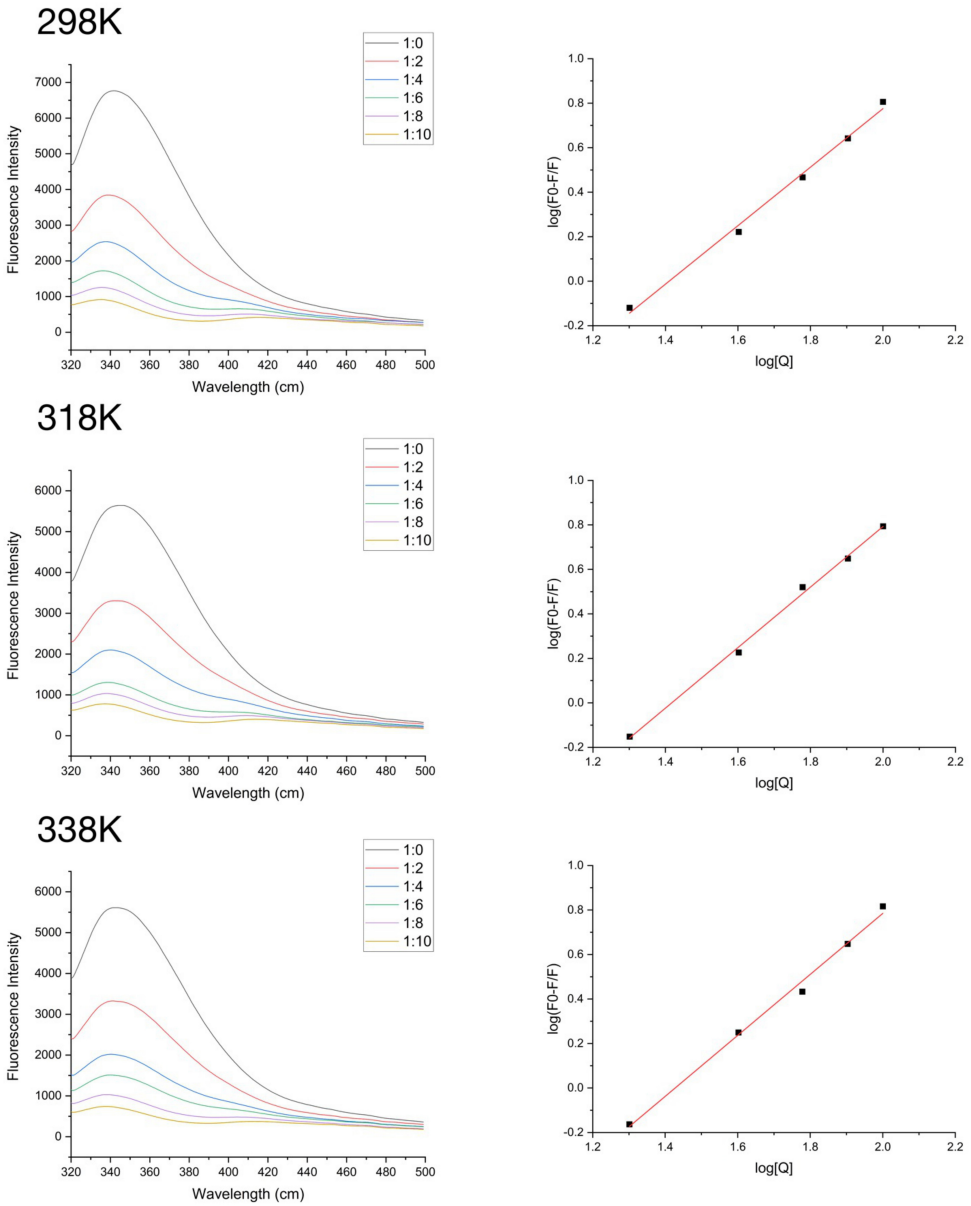
At 298K, 318K, and 338K, the fluorescence intensity of SPI and quercetin (Que) complexes in the range of 320–500 nm and the logarithmic equation fitting curve.

Fluorescence quenching is widely used to study the protein-polyphenol interactions and can provide the detailed information of the combination. The fluorescence quenching pattern of SPI containing different amounts of quercetin are shown in Figure 2. The maximum fluorescence emission wavelength (λ_max_) of SPI is about 330 nm. The fluorescence intensity of SPI decreased significantly (*p* < 0.05) as the concentration of quercetin increased in the system, and the maximum wavelength (λ_max_) showed a blue shift (*p* < 0.05). These phenomena indicated an interaction between SPI and Que, and tryptophan in protein molecule transferred toward hydrophobic environment (40).

To accurately locate the interaction mechanism, the Stern-Volmer equation was used to analyze the data. As shown in Table 1, there was a strong linear relationship between the concentration of quercetin and F_0_/F (*R*^2^ > 0.96) at different temperatures (298K, 318K, 338K). The value of kq was much higher than maximal diffusion collision quenching constant, 2.0 × 10^10^ L•mol^−1^s^−1^ (41) suggesting that the fluorescence quenching induced by quercetin took the form of static quenching (33). According to Table 1, the binding stoichiometry was approximately only 1, manifesting that quercetin interacted with SPI at a 1:1 M ratio regardless of thermal treatments.

**Table 1:** The quenching constants, binding constants, and thermodynamic parameters for SPI-quercetin complexes at 298K, 318K, and 338K.

**T/K**	**KSV/105 L**·**mol**^−1^	**kq/1,014 L**·**mol**^−1^**s**^−1^	**Ka/105 L**·**mol**^−1^	***n***	Δ**H/kJ**·**mol**^−1^	Δ**G/kJ**·**mol**^−1^	Δ**S/kJ**·**mol**^−1^·**K**^−1^
298	0.63 ± 0.60	0.21 ± 0.20	3.06 ± 0.20	1.31 ± 0.06	4.04	−27.25	91.46
318	0.63 ± 0.40	0.21 ± 0.10	2.75 ± 0.22	1.36 ± 0.04		−29.07	91.42
338	0.64 ± 0.70	0.21 ± 0.20	2.83 ± 0.23	1.37 ± 0.06		−31.23	92.41


Small organic molecules always combined with protein to form a supramolecular complex by electrostatic force, hydrogen bonding force, van der Waals force or hydrophobic force. Different interaction forces existed between different molecules. The nature and magnitude of the thermodynamic parameters associated with various individual kinds of molecular interactions have been characterized by Ross and Subramanian (42). The thermodynamic parameters of the combination between quercetin and SPI were shown in Table 1. The calculated values of ΔG were −27.25, −29.07, and −31.23 kJ mol^−1^ at 25, 45 and 65°C, respectively, which revealed the spontaneity binding of quercetin and SPI. The values of ΔH and ΔS were 4.4 kJ mol^−1^ and on average 91.4 J mol^−1^ K^−1^, respectively. According to Ross et al. (42), when ΔH > 0, ΔS > 0, predominant are the hydrophobic interactions; when ΔH <0, ΔS <0, Vander Waals forces and hydrogen bonds dominate; ΔH <0, ΔS > 0 indicates the domination of electrostatic forces. The values of ΔH and ΔS in this study were both positive illustrating that hydrophobic interaction was the primary binding force between quercetin and SPI. The positive value of ΔH was possibly dictated by the following endothermic factors: (1) quercetin molecules partly destroy the hydrophobic hydration structure of SPI, and (2) the original iceberg structure surrounding quercetin molecules is changed, when quercetin molecules insert into the hydrophobic pockets of SPI (43). The value of ΔS was also positive, which might be related to the release of combined water molecules from the molecule pocket, the hydration layer on the surface of the SPI particles and the iceberg structure surrounding the hydrophobic parts of quercetin to buffer medium (29). Furthermore, the value of ΔH was much lower than the interaction energy of the chemical bond (>100 kJ/mol) suggesting that the interaction force between quercetin and SPI was a weak intermolecular force.

### Fourier-transform infrared (FTIR) analysis

**Figure 3:**
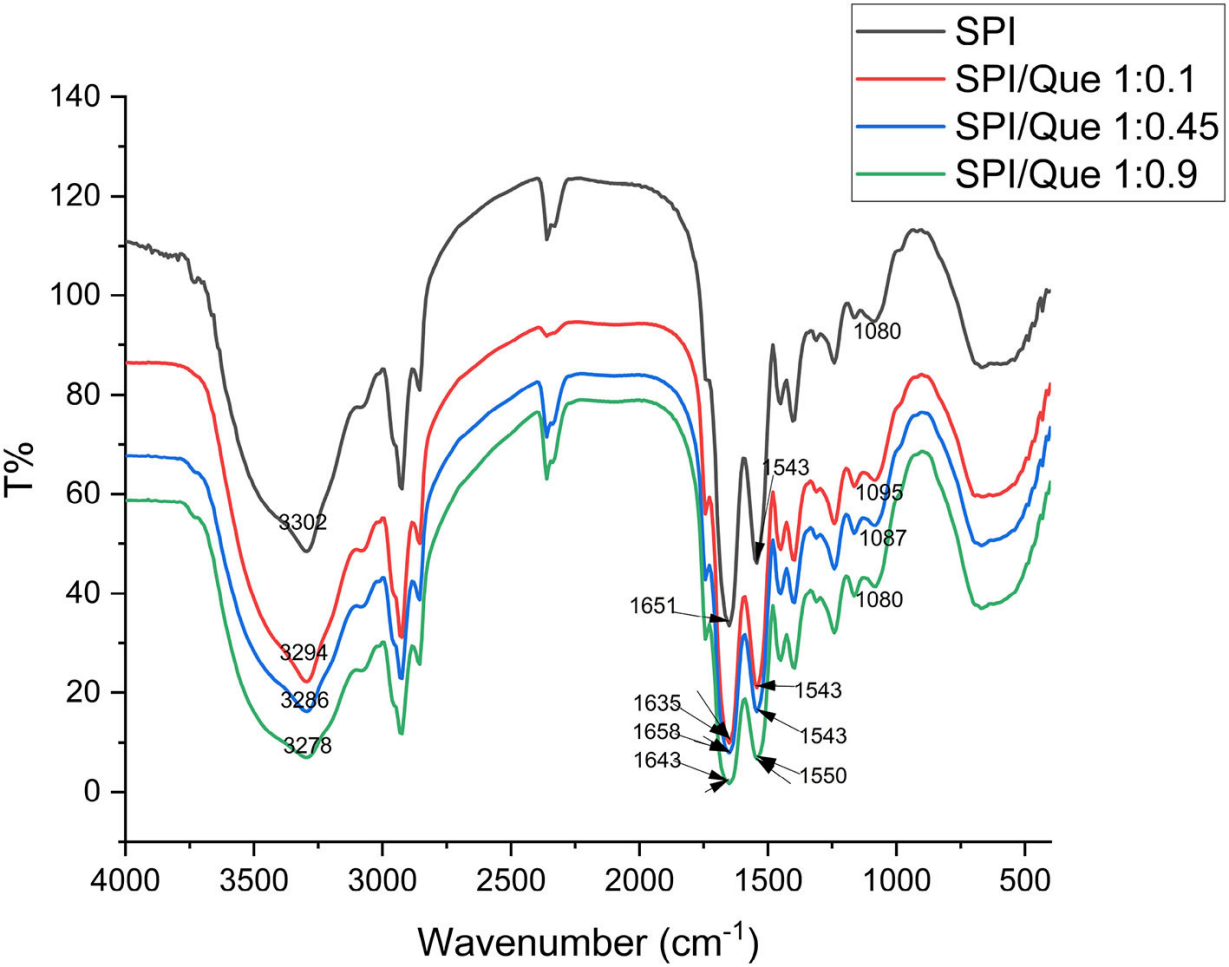
Fourier transform infrared spectra of SPI and SPI-quercetin complexes with different molar ratio (1:0.1, 1:0.45, 1:0.9) at 298K. The corresponding wavelength of the characteristic peaks were marked in this figure.

Structure of proteins can be determined from the infrared spectra of their amide bands. To get further structural change information about SPI before and after the addition of quercetin, the FTIR profiles were recorded in Figure 3.

The broad band around 3,000–3,500 cm^−1^ indicated the O-H or N–H stretching vibration. and the absorption peak showed a significant blue shift with the increasement of quercetin ratio, indicating that SPI was affected through the formation of hydrogen bonds (44). The amide I band located around 1,600–1,700 cm^−1^, mainly relating to C=O stretch. The amide II band located <1,548 cm^−1^, associating with the vibrations of N–H bending and C–N stretching. 1,600 cm^−1^ is the characteristic peak of C=O stretching vibration (45).

Changes were also found with the amide I band located at 1,651 cm^−1^. A significant blue shift in amide I band was observed, and the peak strength increased (Figure 3). This phenomenon might be explained by the fact that the hydrogen bond between SPI and quercetin enhanced with the increasement of quercetin content in the same solvent and temperature conditions. In conclusion, the characteristic peaks of SPI in the range of 4,000–1,000 cm^−1^ showed varying degrees of blue shift, indicating that the secondary structure of SPI changes under the influence of different concentrations of quercetin, which was likely to be provided by hydrogen bonds or hydrophobic interaction (46). In combination with our fluorescence spectrum analysis results, it could be roughly concluded that hydrophobic interaction and hydrogen bonding acted as the main binding forces between SPI and quercetin, which was in accordance with the conclusion of Y. Wang et al. (29).

### Circular dichroism (CD) analysis

**Figure 4:**
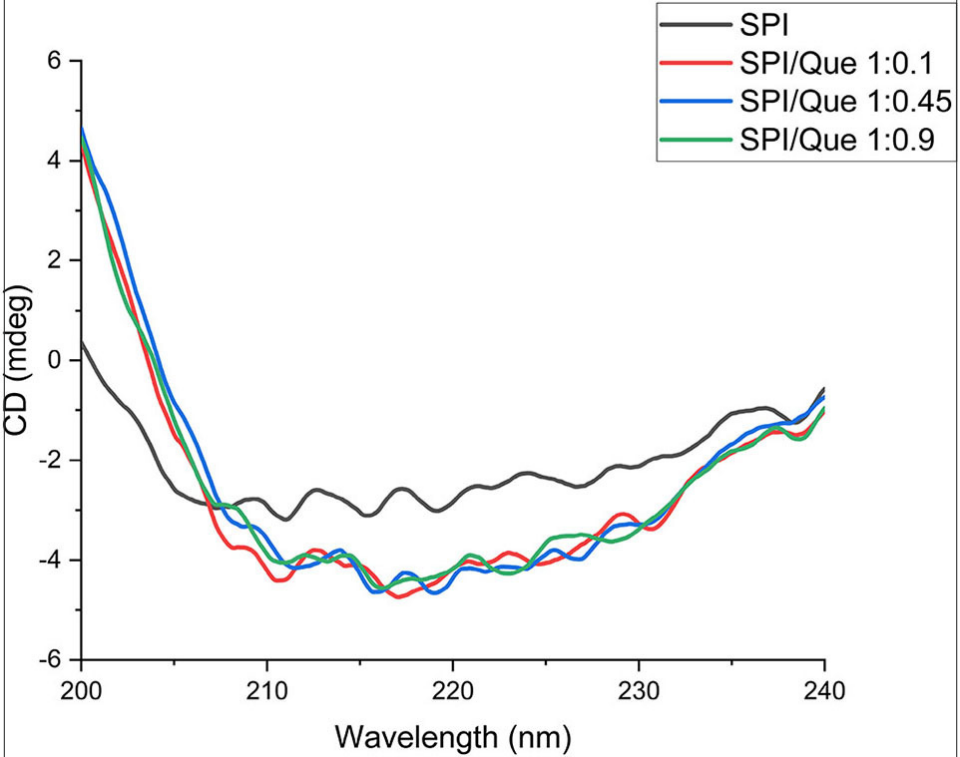
Circular dichroism spectra of SPI and SPI-quercetin complexes with different molar ratios (1:0.1, 1:0.45, 1:0.9) in the wavelength range of 200–240 nm.

CD is considered as a sensitive technique which could monitor the conformational changes of proteins or proteins binding with ligands (47). In this study, circular dichroism was used to characterize the secondary structure changes of SPI in the absence and presence of quercetin with different ratios (Figure 4). The contents of the α-helix, β-sheet, β-turns, and random coils calculated by the CDPro software were listed in Table 2. According to Figure 4, the CD spectra profiles of all samples (Figure 4) showed a broad negative band at around 200–235 nm, suggesting that SPI was rich in random coil structures. The negative ellipticities of SPI around 210–220 nm decreased after quercetin addition at different ratios (*p* < 0.05), revealing that the decrease of α-helix was accompanied by an increase in β-sheet and β-turn compared with that of SPI (Table 2) (33, 35). The result is consistent with previous findings of Katouzian et al about the interaction of bovine α-lactalbumin and oleuropein (48). It could be speculated that the addition of quercetin caused the transition of SPI conformation from order to disorder due to hydrogen bonding and hydrophobic interaction. Consequently, the interaction of SPI-quercetin mixture resulted in the formation of a partially unfolded and more flexible complex, further supporting the conclusion of Section Fourier-Transform Infrared (FTIR) Analysis (49).

**Table 2:** Percentage of different secondary structures in SPI and SPI-quercetin complexes.

**Sample**	**α-helix (%)**	**β-sheet (%)**	**β-turn (%)**	**Random coil (%)**
SPI	20.40	24.60	20.30	34.70
SPI/Quercetin 1:0.1	19.60	25.40	23.90	31.10
S/Quercetin 1:0.45	18.40	29.80	28.40	28.60
S/Quercetin 1:0.9	14.40	32.10	31.40	22.10

### SDS-PAGE analysis of SPI and que

**Figure 5:**
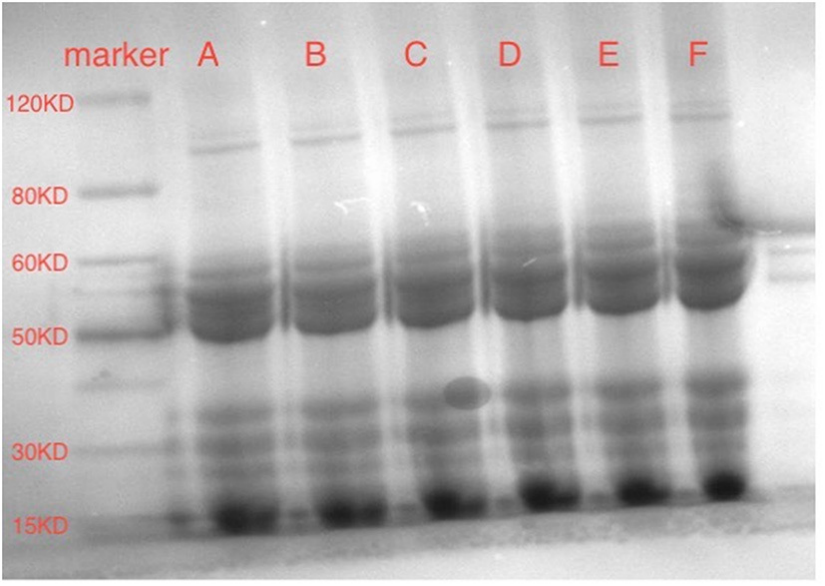
SDS-PAGE of SPI-quercetin complexes: **(A)** SPI sample; **(B)** SPI+20 μM quercetin complex; **(C)** SPI+40 μM quercetin complex; **(D)** SPI+60 μM quercetin complex; **(E)** SPI+80 μM quercetin complex; **(F)** SPI+100 μM Que.

SDS-PAGE analysis was used to monitor the molecular weights of SPI and quercetin mixtures to determine whether a covalent bond formed. As shown in Figure 5, compared with the molecular weight of SPI alone, those of SPI-quercetin mixtures remained almost unchanged with the addition of different amounts of Que. Additionally, there was no new band was observed in Figure 5, indicating there was no covalent bond formed in the complex systems in this study. The results of SDS-PAGE corroborated the conclusion of Section Fluorescence Spectrum Analysis, which revealed that the bonds between SPI and quercetin were mainly non-covalent (50).

### Analysis of molecule docking

**Figure 6:**
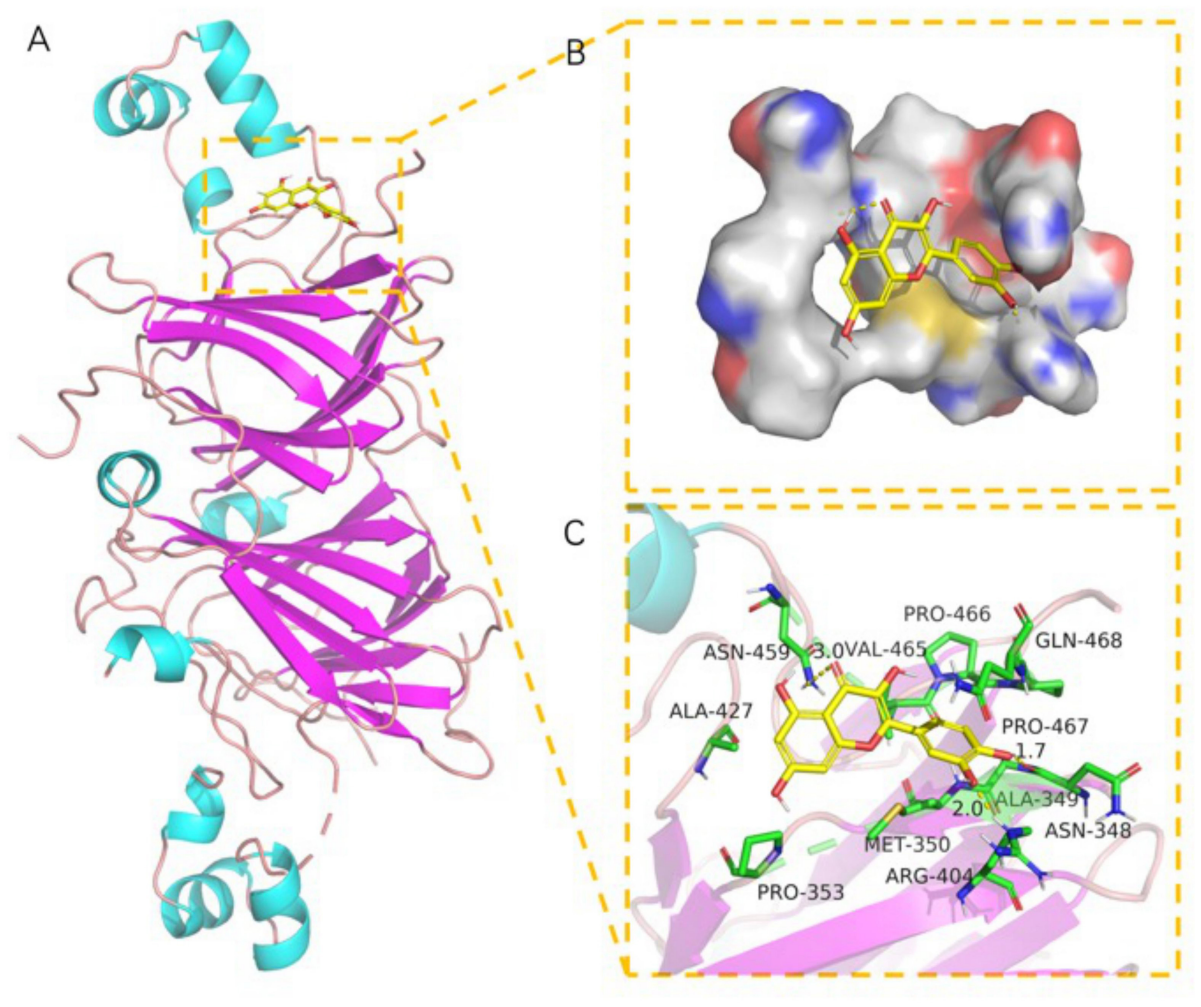
The binding mode of 11S with quercetin: **(A)** The 3D structure of complex. **(B)** The surface of active site. **(C)** The detail binding mode of complex. The backbone of protein was rendered in tube and colored in bright blue. quercetin compound is rendering by yellow.

**Figure 7:**
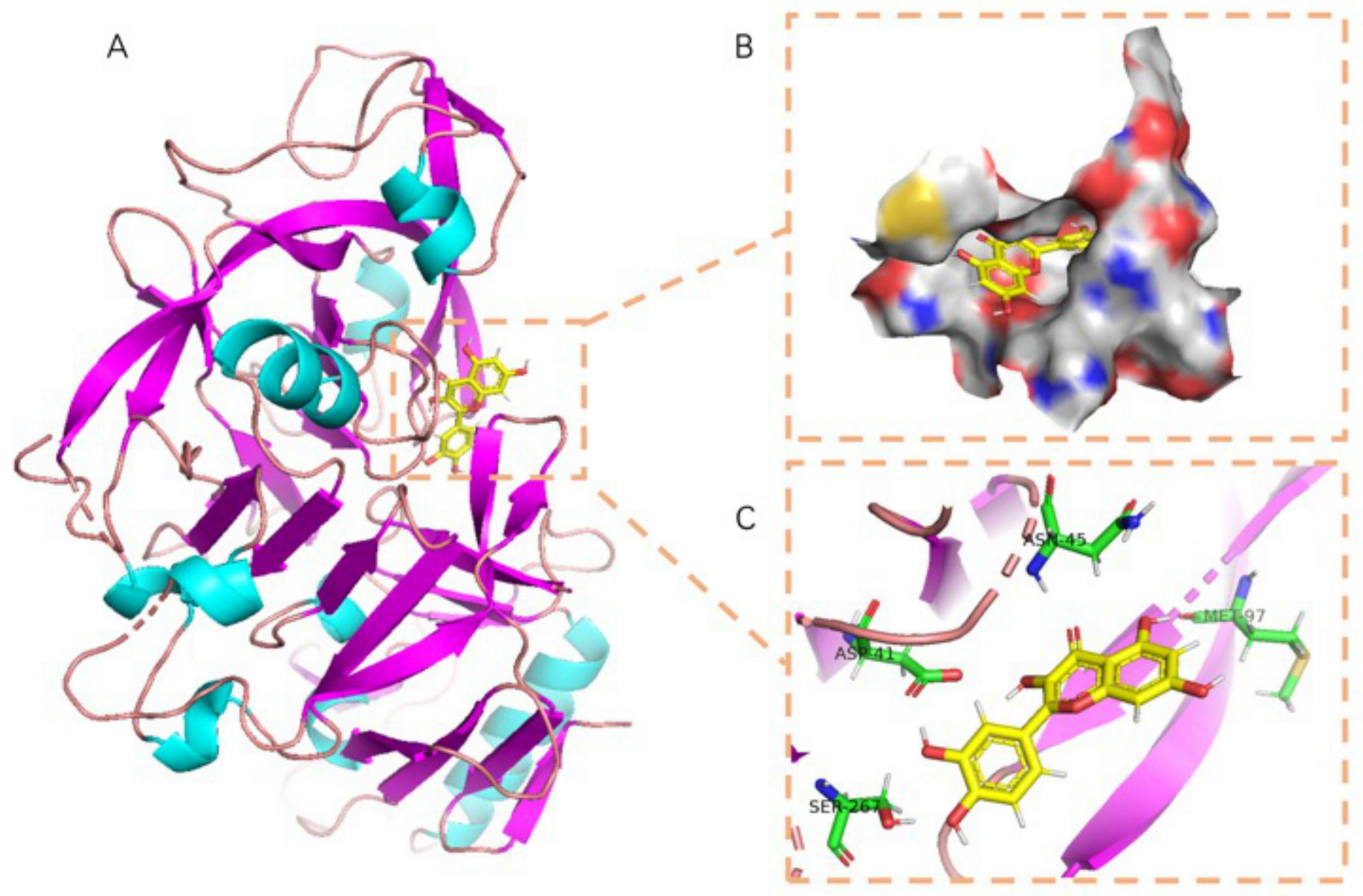
The binding mode of 7S with quercetin: **(A)** The 3D structure of complex. **(B)** The surface of active site. **(C)** The detail binding mode of complex. The backbone of protein and quercetin compound were colored at the same.

SPI contains a variety of globulin components, among which 11S and 7S are the main components (51). The best docking models of 7S (**Figures 7A–C**) and 11S (Figures 6A–C) components of SPI with quercetin were conducted, respectively. The results showed that there were good binding effect and high matching between quercetin and the target proteins with binding energies of −7.25 and −6.38 kcal/mol, respectively (Table 3). Pymol 2.5 software was used to visualize the docking compound and protein complex to determine the binding mode of the two components. In 11S component, the binding of the amino acid and the matching posture of ligands to protein pockets were observed. The active amino acid residues of quercetin were bound to 11S, including ASN-348, ARG-404, PRO-467, ALA-427, PRO-353, MET-350, ALA-349, PRO-467, etc. Several hydrogen bond donor receptors were found in quercetin. The hydrogen bonds with ASN-348, ARG-404, and ARG-459 amino acids were short, testifying the binding ability was strong, which played a crucial role in stabilizing small molecules. Figure 6B also showed that quercetin matched the pocket of 11S globulin very well, and the benzene ring could form a good hydrophobic interaction with the groove.

**Table 3:** The selected compounds of docking results.

**Target ID**	**Compounds**	**Docking Score (kcal/mol)**	**Combination Type**
11S (1fxz)	Quercetin	−7.25	Hydrogen bonds
7S (3aup)	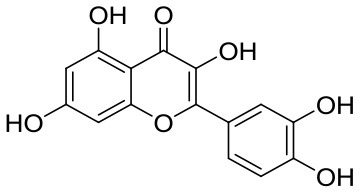	−6.38	Hydrophobic interactive

*Binding energy function (52). :G_bind_ = C_lipo−lipo_∑f(γ_ir_)+C_hbond−neut−neut_∑g(γ)h(α)+C_hbond−charge−charge_∑g(γ)h(α)+C_max−metal−ion_∑f(γ_lm_)+C_rotb_H_robt_+C_polar−phob_V_polar−phob_+C_coul_E_coul_+C_vdW_E_vdW_+solvationterms*.

The active amino acid residues of quercetin binding to 7S globulin were MET-97, ASN-45, ASP-41, SER-267, ARG-356, GLN-104, etc. quercetin could form four hydrogen bonds with MET-97, ASN-45, ASP-41 and SER-267, and the hydrogen bonds distance was short suggesting the bonding ability was strong. According to Figure 7B, quercetin also had a high matching degree with 7S, and the benzene ring could form a good hydrophobic interaction with the protein's central cavity. In summary, quercetin compounds had good binding patterns and docking scores with the active sites of the two main proteins constituting in SPI, which meant quercetin was a potentially active small molecule. Both 11S and 7S had hydrogen bonding and hydrophobic interactions with the quercetin ligand, in agreement with the findings of multiple spectroscopies above.

### Emulsifying properties

**Figure 8:**
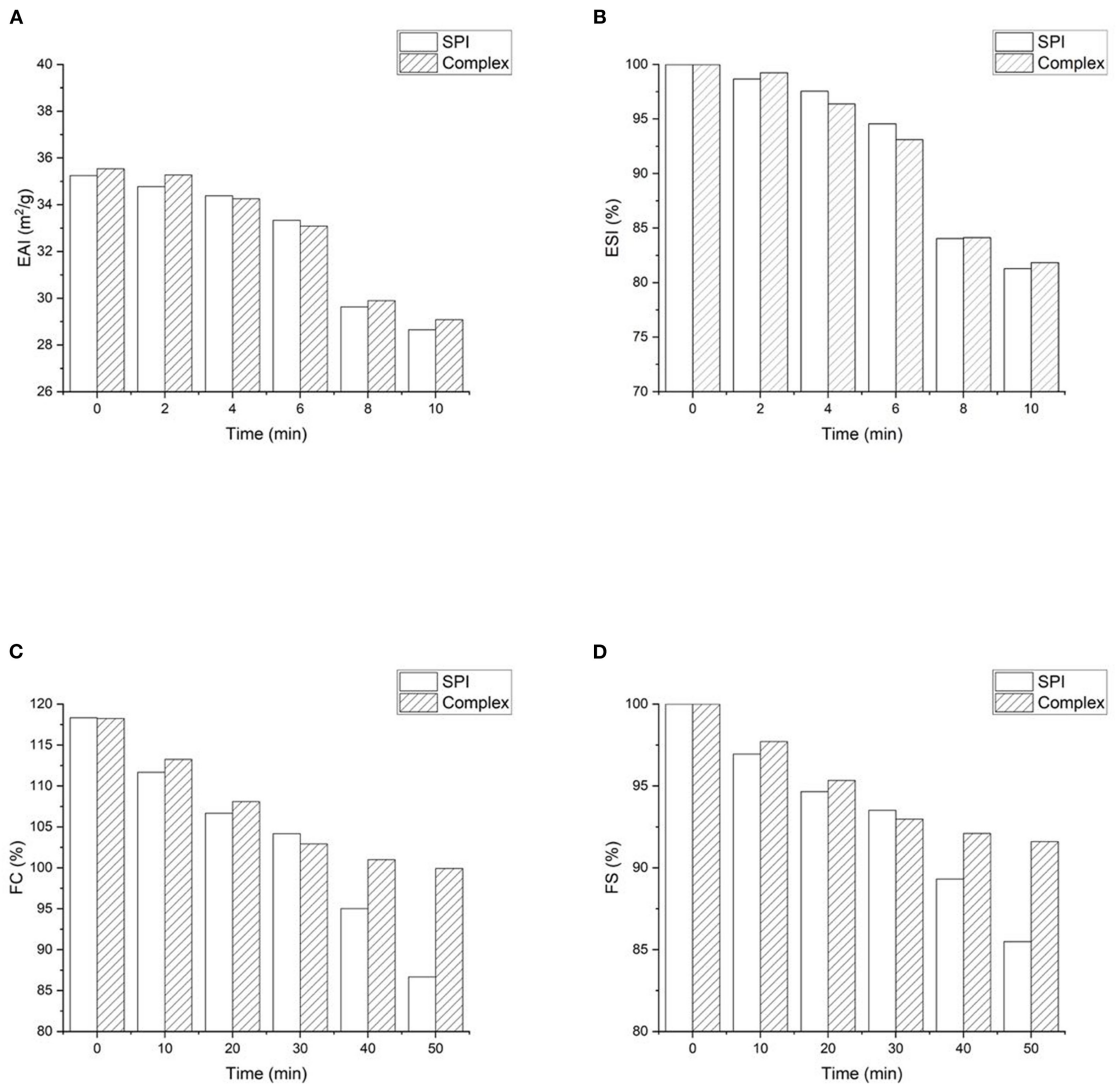
Functional properties of SPI-quercetin complex: **(A)** Foaming capacity (FC); **(B)** foaming stability (FS); **(C)** Emulsifying activity index (EAI); **(D)** emulsifying stability index (ESI).

Emulsifying capacity is the ability to rapidly form a stable interface at the oil-water interface, which is defined as emulsion activity index (EAI). Emulsifying stability (ESI) refers to the ability to maintain the emulsion state and prevent the separation (8). EAI and ESI of SPI and SPI- quercetin complexes are presented in Figures 8A,B. The initial EAI (0 min) of SPI-quercetin increased comparing with that of native SPI, while there was little variation in the ESI. This revealed that modification with quercetin could improve the emulsifying capacity of SPI, which was in consistent with Meng et al. (53), who found the addition of GA, CA, and EGCG increased the EAI comparing to control WPI. However, it was found excess negative zeta potential of polyphenol would hinder proteins to maintain the interface stability due to increased repulsion (54), that might be the reason for the change of ESI.

### Foaming properties

The foaming behavior of SPI was related to the rapidly adsorption and film forming capacity at the air-water interface (53). The foaming capacity (FC) and foaming stability (FS) of SPI and SPI-quercetin complexes are shown in Figures 8C,D. Both the FC and FS of SPI showed a slight increase after the addition of quercetin within 30 min, while a rapid increase was observed at 30 min and 40 min. According to the conformational analysis above, protein with partially unfolded and more flexible structure could be more effective to diffuse to the air-water interface and reduce the surface tension of the air bubbles, which improved the foaming behavior (55). In addition, Morfo Zembyla confirmed that the stabilized system was also probably attribute to the electrostatic attraction between the oppositely-charged polyphenol particles and protein at the interface, although a more order and stable structure was induced by quercetin (56).

## Conclusion

In this work, conformational changes and functional properties of SPI modified by quercetin has been investigated. Fluorescence data demonstrated that quercetin induced the static quenching on the fluorescence activity of SPI, leading to the structure changes of SPI, which was confirmed by CD spectroscopy and FTIR results. Further analysis showed that quercetin binded strongly with SPI to form a more flexible structure, primarily through hydrogen bonding and hydrophobic forces with the binding stoichiometry approximately equal to 1. After binding with quercetin, the structure of SPI became looser and flexible, which led to an improvement of its emulsifying and foaming properties. Although more investigation needs to be conducted, these findings in this study were expected to serve as a theoretical reference for the development of novel plant protein-polyphenols complex products and their application in the food industry in future.

## Data availability statement

The original contributions presented in the study are included in the article/Supplementary Material, further inquiries can be directed to the corresponding author/s.

## Author contributions

YZ: funding acquisition, project administration, supervision, writing and editing, and resources. RH: data curation, writing-original draft, investigation, and validation. WZ: investigation, data curation, writing, methodology, and validation. BZ: resources, conceptualization, and supervision. JuZ: investigation and validation. GY and JiZ: project administration. TL and ZZ: investigation. HW: funding acquisition, project administration, and supervision. ZL: funding acquisition, resources, and supervision. All authors contributed to the article and approved the submitted version.

## Funding

This research was supported by the National Natural Science Foundation of China (NSFC32001698).

## Conflict of interest

The authors declare that the research was conducted in the absence of any commercial or financial relationships that could be construed as a potential conflict of interest.

## Publisher's note

All claims expressed in this article are solely those of the authors and do not necessarily represent those of their affiliated organizations, or those of the publisher, the editors and the reviewers. Any product that may be evaluated in this article, or claim that may be made by its manufacturer, is not guaranteed or endorsed by the publisher.

## References

[1] Zhang Y Tan C Eric K Abbas S Liu F Zhang X. Effect of limited enzymatic hydrolysis on physico-chemical properties of soybean protein isolate-maltodextrin conjugates. Int J Food Sci Technol. (2015) 50:226–32. 10.1111/ijfs.12624

[2] Zhong MM Sun YF Sun YD Fang L Qi BK Xie FY. Dynamic gastric stability and *in vitro* lipid digestion of soybean protein isolate and three storage protein-stabilized emulsions: effects of ultrasonic treatment. Food Res Int. (2021) 149:110666. 10.1016/j.foodres.2021.110666 34600668

[3] Liang G Chen W Qie X Zeng M Qin F He Z. Modification of soy protein isolates using combined pre-heat treatment and controlled enzymatic hydrolysis for improving foaming properties. Food Hydrocolloids. (2020) 105:105764. 10.1016/j.foodhyd.2020.105764

[4] Ma X Chi C Pu Y Miao S Liu D. Conjugation of soy protein isolate (SPI) with pectin: effects of structural modification of the grafting polysaccharide. Food Chem. (2022) 387:132876. 10.1016/j.foodchem.2022.132876 35395480

[5] Wang J Zhao M Qiu C Sun W. Effect of malondialdehyde modification on the binding of aroma compounds to soy protein isolates. Food Res Int. (2018) 105:150–58. 10.1016/j.foodres.2017.11.001 29433202

[6] Shen P Zhou F Zhang Y Yuan D Zhao Q Zhao M. Formation and characterization of soy protein nanoparticles by controlled partial enzymatic hydrolysis. Food Hydrocolloids. (2020) 105:105844. 10.1016/j.foodhyd.2020.105844

[7] Jiang J Zhang ZP Zhao J Liu YF. The effect of non-covalent interaction of chlorogenic acid with whey protein and casein on physicochemical and radical-scavenging activity of *in vitro* protein digests. Food Chem. (2018) 268:334–41. 10.1016/j.foodchem.2018.06.015 30064766

[8] You Y Yang L Chen H Xiong L Yang F. Effects of (-)-Epigallocatechin-3-gallate on the functional and structural properties of soybean protein isolate. J Agric Food Chem. (2021) 69:2306–15. 10.1021/acs.jafc.0c07337 33576221

[9] Ulusoy HG Sanlier N. A minireview of quercetin: from its metabolism to possible mechanisms of its biological activities. Crit Rev Food Sci Nutr. (2020) 60:3290–303. 10.1080/10408398.2019.1683810 31680558

[10] Yang Y Wang Q Lei L Li F Zhao J Zhang Y. Molecular interaction of soybean glycinin and beta-conglycinin with (-)-epigallocatechin gallate induced by pH changes. Food Hydrocolloids. (2020) 108:106010. 10.1016/j.foodhyd.2020.106010

[11] Lee GH Lee SJ Jeong SW Kim HC Park GY Lee SG. Antioxidative and antiinflammatory activities of quercetin-loaded silica nanoparticles. Colloids Surf B Biointerfaces. (2016) 143:511–17. 10.1016/j.colsurfb.2016.03.060 27038916

[12] Geng LL Liu ZP Zhang WQ Li W Wu ZM Wang W. Chemical screen identifies a geroprotective role of quercetin in premature aging. Protein Cell. (2019) 10:417–35. 10.1007/s13238-018-0567-y 30069858 PMC6538594

[13] Faraji S Nowroozi N Nouralishahi A Shabani Shayeh J. Electrospun poly-caprolactone/graphene oxide/quercetin nanofibrous scaffold for wound dressing: evaluation of biological and structural properties. Life Sci. (2020) 257:118062. 10.1016/j.lfs.2020.118062 32652138

[14] He Z Zhang X Song Z Li L Chang H Li S. Quercetin inhibits virulence properties of *Porphyromas gingivalis* in periodontal disease. Sci Rep. (2020) 10:18313. 10.1038/s41598-020-74977-y 33110205 PMC7591570

[15] Diao Y Yu X Zhang C Jing Y. Quercetin-grafted chitosan prepared by free radical grafting: characterization and evaluation of antioxidant and antibacterial properties. J Food Sci Technol. (2020) 57:2259–68. 10.1007/s13197-020-04263-2 32431352 PMC7230106

[16] Moon H Lertpatipanpong P Hong Y Kim CT Baek SJ. Nano-encapsulated quercetin by soluble soybean polysaccharide/chitosan enhances anti-cancer, anti-inflammation, anti-oxidant activities. J Funct Foods. (2021) 87:104756. 10.1016/j.jff.2021.104756

[17] Sadighparvar S Darband SG Yousefi B Kaviani M Ghaderi-Pakdel F Mihanfar A. Combination of quercetin and exercise training attenuates depression in rats with 1,2-dimethylhydrazine-induced colorectal cancer: possible involvement of inflammation and BDNF signalling. Exp Physiol. (2020) 105:1598–609. 10.1113/EP088605 32681548

[18] Wang J Zhao XH. Degradation kinetics of fisetin and quercetin in solutions affected by medium pH, temperature and co-existing proteins. J Serbian Chem Soc. (2016) 81:243–53. 10.2298/JSC150706092W

[19] Doosti M Dorraji MSS Mousavi SN Rasoulifard MH Hosseini SH. Enhancing quercetin bioavailability by super paramagnetic starch-based hydrogel grafted with fumaric acid: an *in vitro* and *in vivo* study. Colloids Surf B Biointerfaces. (2019) 183:110487. 10.1016/j.colsurfb.2019.110487 31518957

[20] Manzoor MF Hussain A Sameen A Sahar A Khan S Siddique R. Novel extraction, rapid assessment and bioavailability improvement of quercetin: a review. Ultrasonics Sonochem. (2021) 78:105686 . 10.1016/j.ultsonch.2021.105686 PMC8350193 34358980

[21] Chen LC Chen YC Su CY Hong CS Ho HO Sheu MT. Development and characterization of self-assembling lecithin-based mixed polymeric micelles containing quercetin in cancer treatment and an *in vivo* pharmacokinetic study. Int J Nanomed. (2016) 11:1557–66. 10.2147/IJN.S103681 27143878 PMC4841422

[22] Wiczkowski W Szawara-Nowak D Topolska J. Red cabbage anthocyanins: profile, isolation, identification, antioxidant activity. Food Res Int. (2013) 51:303–9. 10.1016/j.foodres.2012.12.015

[23] Betz M Kulozik U. Microencapsulation of bioactive bilberry anthocyanins by means of whey protein gels. In: 11th International Congress on Engineering and Food (ICEF). Athens (2011). pp. 2047–56.

[24] Wang Q Wei H Deng C Xie C Huang M Zheng F. Improving stability and accessibility of quercetin in olive oil-in-soy protein isolate/pectin stabilized O/W emulsion. Foods. (2020) 9:123. 10.3390/foods9020123 31979401 PMC7073632

[25] Papakyriakopoulou P Manta K Kostantini C Kikionis S Banella S Ioannou E. Nasal powders of quercetin-beta-cyclodextrin derivatives complexes with mannitol/lecithin microparticles for Nose-to-Brain delivery: *in vitro* and *ex vivo* evaluation. Int J Pharm. (2021) 607:121016. 10.1016/j.ijpharm.2021.121016 34411652

[26] Li Y Gao S Ji X Liu H Liu N Yang J. Evaluation studies on effects of quercetin with different concentrations on the physicochemical properties and *in vitro* digestibility of Tartary buckwheat starch. Int J Biol Macromol. (2020) 163:1729–37. 10.1016/j.ijbiomac.2020.09.116 32979438

[27] Xu JJ Guo SY Li XJ Jiang ST Zhong XY Zheng Z. Gel properties of transglutaminase-induced soy protein isolate-polyphenol complex: influence of epigallocatechin-3-gallate. J Sci Food Agric. (2021) 101:3870–9. 10.1002/jsfa.11025 33336789

[28] Yang YX Wang QM Tang YW Lei L Zhao JC Zhang YH. Effects of ionic strength and (-)-epigallocatechin gallate on physicochemical characteristics of soybean 11S and 7S proteins. Food Hydrocolloids. (2021) 119:106836. 10.1016/j.foodhyd.2021.106836

[29] Wang Y Wang X. Binding, stability, and antioxidant activity of quercetin with soy protein isolate particles. Food Chem. (2015) 188:24–9. 10.1016/j.foodchem.2015.04.127 26041159

[30] Ao L Liu P Wu A Zhao J Hu X. Characterization of soybean protein isolate-food polyphenol interaction via virtual screening and experimental studies. Foods. (2021) 10:2813. 10.3390/foods10112813 34829094 PMC8625844

[31] Li Y Liu B Jiang L Regenstein JM Jiang N Poias V. Interaction of soybean protein isolate and phosphatidylcholine in nanoemulsions: a fluorescence analysis. Food Hydrocolloids. (2019) 87:814–29. 10.1016/j.foodhyd.2018.09.006

[32] Liu K Zha XQ Shen WD Li QM Pan LH Luo JP. The hydrogel of whey protein isolate coated by lotus root amylopectin enhance the stability and bioavailability of quercetin. Carbohydr Polym. (2020) 236:116009. 10.1016/j.carbpol.2020.116009 32172837

[33] Yin Z Qie X Zeng M Wang Z Qin F Chen J. Effect of thermal treatment on the molecular-level interactions and antioxidant activities in β-casein and chlorogenic acid complexes. Food Hydrocolloids. (2022) 123:107177. 10.1016/j.foodhyd.2021.107177

[34] Wei J Xu D Yang J Zhang X Mu T Wang Q. Analysis of the interaction mechanism of Anthocyanins (*Aronia melanocarpa* Elliot) with β-casein. Food Hydrocolloids. (2018) 84:276–281. 10.1016/j.foodhyd.2018.06.011

[35] Condict L Kaur J Hung A Ashton J Kasapis S. Combined spectroscopic, molecular docking and quantum mechanics study of β-casein and ferulic acid interactions following UHT-like treatment. Food Hydrocolloids. (2019) 89:351–9. 10.1016/j.foodhyd.2018.10.055

[36] Wu X Lu Y Xu H Lin D He Z Wu H. Reducing the allergenic capacity of beta-lactoglobulin by covalent conjugation with dietary polyphenols. Food Chem. (2018) 256:427–34. 10.1016/j.foodchem.2018.02.158 29606470

[37] Fazi R Tintori C Brai A Botta L Selvaraj M Garbelli A. Homology model-based virtual screening for the identification of human helicase DDX3 inhibitors. J Chem Inform Model. (2015) 55:2443–54. 10.1021/acs.jcim.5b00419 26544088

[38] Rajeswari M Santhi N Bhuvaneswari V. Pharmacophore and virtual screening of JAK3 inhibitors. Bioinformation. (2014) 10:157–63. 10.6026/97320630010157 24748756 PMC3974243

[39] Antonio E Khalil NM Mainardes RM. Bovine serum albumin nanoparticles containing quercetin: characterization and antioxidant activity. J Nanosci Nanotechnol. (2016) 16:1346–53. 10.1166/jnn.2016.11672 27433585

[40] Cui Z Kong X Chen Y Zhang C Hua Y. Effects of rutin incorporation on the physical and oxidative stability of soy protein-stabilized emulsions. Food Hydrocolloids. (2014) 41:1–9. 10.1016/j.foodhyd.2014.03.006

[41] Lakowicz JR Weber G. Quenching of fluorescence by oxygen - probe for structural fluctuations in macromolecules. Biochemistry. (1973) 12:4161–70. 10.1021/bi00745a020 4795686 PMC6959846

[42] Ross PD Subramanian S. Thermodynamics of protein association reactions: forces contributing to stability. Biochemistry. (1981) 20:3096–102. 10.1021/bi00514a017 7248271

[43] Li X Chen D Wang G Lu Y. Study of interaction between human serum albumin and three antioxidants: ascorbic acid, alpha-tocopherol, and proanthocyanidins. Eur J Med Chem. (2013) 70:22–36. 10.1016/j.ejmech.2013.09.033 24140914

[44] Seczyk L Swieca M Kapusta I Gawlik-Dziki U. Protein-phenolic interactions as a factor affecting the physicochemical properties of white bean proteins. Molecules. (2019) 24:408. 10.3390/molecules24030408 30678067 PMC6384846

[45] Wang QM Tang YW Yang YX Zhao JC Zhang YH Li L. Interaction between wheat gliadin and quercetin under different pH conditions analyzed by multi-spectroscopy methods. Spectrochim Acta Part A Mol Biomol Spectrosc. (2020) 229:117937. 10.1016/j.saa.2019.117937 31865099

[46] Liu FG Ma CC McClements DJ Gao YX. A comparative study of covalent and non-covalent interactions between zein and polyphenols in ethanol-water solution. Food Hydrocolloids. (2017) 63:625–34. 10.1016/j.foodhyd.2016.09.041

[47] Kelly SM Jess TJ Price NC. How to study proteins by circular dichroism. Biochim Biophys Acta Proteins Proteomics. (2005) 1751:119–39. 10.1016/j.bbapap.2005.06.005 16027053

[48] Katouzian I Jafari SM Maghsoudlou Y Karami L Eikani MH. Experimental and molecular docking study of the binding interactions between bovine α-lactalbumin and oleuropein. Food Hydrocolloids. (2020) 105:105859. 10.1016/j.foodhyd.2020.105859

[49] Chen Z Wang C Gao X Chen Y Kumar Santhanam R Wang C. Interaction characterization of preheated soy protein isolate with cyanidin-3-O-glucoside and their effects on the stability of black soybean seed coat anthocyanins extracts. Food Chem. (2019) 271:266–73. 10.1016/j.foodchem.2018.07.170 30236676

[50] Zhang Y-J Zhang N Zhao X-H. The non-covalent interaction between two polyphenols and caseinate as affected by two types of enzymatic protein crosslinking. Food Chem. (2021) 364:130375. 10.1016/j.foodchem.2021.130375 34167009

[51] Chen G Wang S Feng B Jiang B Miao M. Interaction between soybean protein and tea polyphenols under high pressure. Food Chem. (2019) 277:632–8. 10.1016/j.foodchem.2018.11.024 30502197

[52] Friesner RA Banks JL Murphy RB Halgren TA Klicic JJ Mainz DT. Glide: a new approach for rapid, accurate docking and scoring. 1. Method and assessment of docking accuracy. J Med Chem. (2004) 47:1739–49. 10.1021/jm0306430 15027865

[53] Meng Y Li C. Conformational changes and functional properties of whey protein isolate-polyphenol complexes formed by non-covalent interaction. Food Chem. (2021) 364:129622. 10.1016/j.foodchem.2021.129622 34175622

[54] Acosta-Dominguez L Cocotle-Ronzon Y Alamilla-Beltran L Hernandez-Martinez E. Effect of a cryogenic treatment in the microstructure, functional and flow properties of soy protein isolate. Food Hydrocolloids. (2021) 119:106871. 10.1016/j.foodhyd.2021.106871

[55] Suib X Sunb H Qi B Zhang M Lib Y Jiang L. Functional and conformational changes to soy proteins accompanying anthocyanins: focus on covalent and non-covalent interactions. Food Chem. (2018) 245:871–8. 10.1016/j.foodchem.2017.11.090 29287453

[56] Zembyla M Murray BS Radford SJ Sarkar A. Water-in-oil Pickering emulsions stabilized by an interfacial complex of water-insoluble polyphenol crystals and protein. J Colloid Interface Sci. (2019) 548:88–99. 10.1016/j.jcis.2019.04.010 30981966

