# Supplementary figures and images for: Changes on the conformational and functional properties of soybean protein isolate induced by quercetin
Source: Front Nutr. 2022 Jul 22;9:966750. doi: 10.3389/fnut.2022.966750 (PMC9354261; doi:10.3389/fnut.2022.966750)

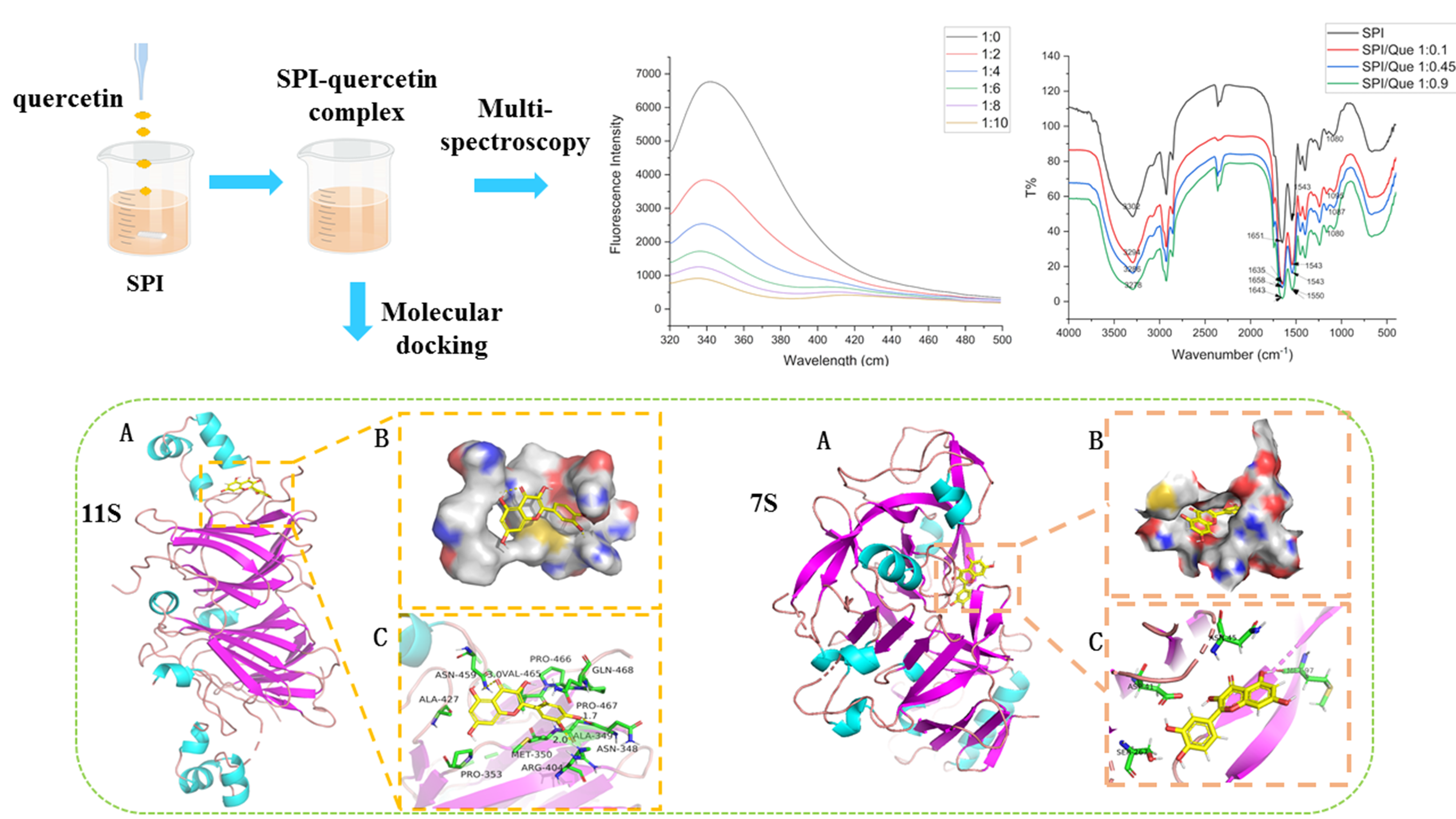

Supplement: Supplementary file 1 [file Image_1.TIF]
